# Pediatric SJS-TEN: Where are we now?

**DOI:** 10.12688/f1000research.20419.1

**Published:** 2020-08-13

**Authors:** Michele Ramien, Jennifer L. Goldman

**Affiliations:** 1 Department of Pediatrics, University of Calgary, Alberta Children's Hospital, 28 Oki Dr NW, Calgary, AB, Canada; 2 Department of Medicine, University of Calgary, Calgary, AB, Canada; 3 Division of Clinical Pharmacology, Toxicology & Therapeutic Innovation, Children’s Mercy Hospitals and Clinics, Kansas City, MO, USA; 4 Division of Pediatric Infectious Diseases, Children’s Mercy Hospitals and Clinics, Kansas City, MO, USA

**Keywords:** Stevens-Johnson syndrome, toxic epidermal necrolysis, severe cutaneous drug reaction, drug reaction, drug eruption, SJS, TEN, reactive infectious mucocutaneous eruption, RIME, drug-induced epidermal necrolysis, DEN

## Abstract

Stevens–Johnson syndrome and toxic epidermal necrolysis are rare severe blistering skin reactions triggered by medications or infections. Over the last 5 to 10 years, a number of important publications have advanced understanding of these diseases and their response to treatment. Importantly, a subset of patients with disease triggered by infection has been identified as having Mycoplasma pneumoniae–induced rash and mucositis, suggesting a reconsideration of the diagnostic paradigm. We present an update on pediatric Stevens–Johnson syndrome and toxic epidermal necrolysis in the broader context of cutaneous adverse drug reactions and focus on challenges and recent advances in diagnosis, management, and prevention.

## Introduction

Skin (cutaneous) reactions are among the most common types of adverse reactions to medications in children, accounting for 36% of any adverse drug reaction (ADR) ^1^. Many of these reactions are mild and self-resolving, but the rare severe ADRs can be associated with significant morbidity and even mortality. The diagnosis and treatment of cutaneous ADRs (cADRs) in children are challenging for several reasons. First, children are more commonly infected with viruses as compared with adults; each year, children in their first years of life average six to 10 respiratory viral infections and older children and adolescents average three to five such illnesses ^2^. Many of these viruses, such as Epstein–Barr virus, adenovirus, and enteroviruses, are frequently associated with cutaneous reactions that can be misinterpreted as cADRs. Second, triggers of severe cADRs, such as Stevens–Johnson syndrome (SJS), are different in adults and children; medications more often are implicated in adults and infections more commonly cause SJS in children ^3^. Third, causality tools currently available—for example, Naranjo, algorithm of drug causality for epidermal necrolysis (ALDEN), and the US Food and Drug Administration’s division of drug experience—have been developed and studied primarily in adults and not applied to children. Finally, owing to the overall rarity of severe cADRs in children, there is little evidence to guide treatment.

ADRs can be either type A (predictable, caused by on-target drug action) or type B (heterogeneous, immunologically and metabolically mediated) ( Table 1). cADRs can be classified clinically by morphology and severity on the basis of the presence (complex) or absence (simple) of fever and other systemic symptoms ( Table 2).

**Table 1.** Cutaneous adverse drug reactions mechanisms summary.

Type A - Augmented	Type B - Bizarre				
Predictable (example: overdose)	Non-immunological (example: intolerance)	Immunological			
		Type I: IgE	Type II: IgG cytotoxic	Type III: immune complexes	Type IV: delayed hypersensitivity
		Urticaria/Angioedema	Hemolytic anemia	Vasculitis	Delayed
					IVa: ACD
					IVb: DiHS/DRESS
					IVc: SJS-TEN, morbilliform
					IVd: AGEP

ACD, allergic contact dermatitis; AGEP, acute generalized exanthematous pustulosis; DiHS, drug-induced hypersensitivity syndrome; DRESS, drug reaction with eosinophilia and systemic symptoms; SJS-TEN, Stevens–Johnson syndrome–toxic epidermal necrolysis ^4^. Adapted with permission from Noguera-Morel *et al*. ^1^.

**Table 2.** Morphology and severity summary.

	Exanthematous	Urticarial	Blistering	Pustular
Simple – no fever	Morbilliform	Urticaria	FDE, SDRIFE	Acneiform
Complex – fever	Drug HSS/DRESS	SSLR	SJS-TEN	AGEP

AGEP, acute generalized exanthematous pustulosis; DRESS, drug reaction with eosinophilia and systemic symptoms; FDE, fixed drug eruption; HSS, hypersensitivity syndrome; SDRIFE, symmetrical drug related intertriginous and flexural exanthem; SJS-TEN, Stevens–Johnson syndrome–toxic epidermal necrolysis; SSLR, serum sickness-like reaction. ^5^.

Given their rarity, complex or severe cADRs are difficult to study systematically and pediatric-specific data are limited. These challenges have led to the adoption of adult paradigms for diagnosis and management in pediatric practice, where better evidence exists.

In SJS and toxic epidermal necrolysis (TEN), severe blistering of the skin and mucous membranes related to either medications or infections occurs. The differential diagnosis in early cases where diffuse erythema is common may include viral exanthems, Kawasaki disease, and acute generalized exanthematous pustulosis. As widespread blistering begins to develop, thermal burns, toxic erythema of chemotherapy, pemphigus of all types, staphylococcal scalded skin syndrome (which spares the mucous membranes), acute graft-versus-host disease, acute syndrome of apoptotic pan-epidermolysis associated with systemic lupus erythematosus, and generalized bullous fixed drug eruption become the primary considerations.

In recent years, significant developments have occurred in the category of severe blistering drug reactions—SJS and TEN—the focus of this review. The watershed evolutions in this area will be reviewed and ultimately support the need for evolution of the diagnostic categories for severe blistering cADRs in children.

## Incidence

Until 2017, estimates of the incidence of SJS and TEN were based on retrospective surveys and databases from the 1990s and a European registry ^6– 9^. More recently, larger datasets have been used in attempts to better describe these rare events in pediatrics.

A recent US pediatric database cohort study suggests that SJS-TEN spectrum disorders occur in 7.5 per 100,000 hospitalized children ^10^; incidences of 6.3 and 0.5 per 100,000 hospitalized children per year for SJS and TEN, respectively, were reported. A second US cross-sectional study that sourced data from the same 2009–2012 time frame found much lower rates, of 5.3 (SJS) and 0.4 (TEN) per million children per year, in the overall population ^11^. Similar findings in both studies suggest that pediatric SJS-TEN results in a substantial health-care burden, although mortality is less when compared with adult data. There are many limitations to using large datasets to examine rare events such as SJS-TEN, including poor validity of International Classification of Diseases 9/10 (ICD-9/10) codes, inability to determine cause, and the lack of a standardized diagnostic approach across institutions. Given that the clinical features of severe cADRs can be challenging to interpret for diagnosis, standardization in case identification and validation is needed.

## Clinical features and refining diagnosis: drug versus bug

### Stevens–Johnson syndrome and toxic epidermal necrolysis

SJS and TEN are characterized by blistering of the skin and mucous membranes. One to three days before onset of skin and mucosal lesions, prodromal symptoms start; these include fever, general malaise, non-productive cough, stinging eyes, and a sore mouth. These symptoms are often mistaken for an upper respiratory tract infection. Macules with purpuric, non-blanching centers with a predilection for the head and torso evolve quickly, often within 12 hours, into blisters that slough off, leaving large areas of denuded skin and mucosa. Painful erythema of the palms and soles is also common early in the disease. Target papules with three distinct rings, characteristic of erythema multiforme, a similar condition usually triggered by herpes simplex virus ^12^, are not the main morphology in SJS and TEN, although atypical two-ringed or macular (flat) targets may occur early, before blistering starts. Mucosal involvement affects oral, ocular, genitourinary, and anal sites.

SJS and TEN are believed to exist on a spectrum; there is less than 10% body surface area (BSA) involvement in SJS and greater than 30% BSA involvement in TEN, and intermediate BSA involvement of 10 to 30% is called SJS-TEN overlap ( Table 3) ^13^. Involved areas include skin that is already blistered or detached and skin that is red (macular erythema) and detachable.

**Table 3.** Severe blistering cutaneous adverse drug reactions and *Mycoplasma pneumoniae*–induced rash and mucositis.

	SJS	SJS-TEN overlap	TEN
Body surface area affected	<10%	10–30%	>30%
	MIRM (usually <10% but can be more extensive)		

MIRM, *Mycoplasma pneumoniae*–induced rash and mucositis; SJS, Stevens–Johnson syndrome; TEN, toxic epidermal necrolysis.

These diagnostic categories were developed in 1993 on the basis of an expert review and synthesis of hundreds of adult cases ^13^, and for many pediatric cases, a diagnostic category can be assigned. However, problems arise when patients have severe involvement of their mucous membranes but little or no skin lesions because there is no diagnostic category for them.

### *Mycoplasma pneumoniae*–induced rash and mucositis

In 2015, Canavan *et al.* ^14^ described *Mycoplasma pneumoniae* (MP)-induced rash and mucositis (MIRM) as an entity distinct from SJS. Patients with MIRM have exactly the features of the previously noted patients who evaded classification; they have severe mucositis of multiple mucous membranes out of proportion to skin involvement, which typically is sparse but in some cases may be significant ^14^. The characteristic constellation of features in MIRM is triggered by respiratory infection rather than medications, and pathogens other than MP have been reported ^15^. Advances in technology, including respiratory polymerase chain reaction (PCR), have improved MP detection, and more specific methods to confirm infection have recently been developed ^16^; these include measurement of MP-specific antibody-secreting cells ^17^.

The challenge with cases attributed to infection and with the diagnosis of MIRM is that their clinical features overlap with those of SJS, creating opportunities for patients to receive multiple diagnoses and complicating more comprehensive and systematic study of these cases in the future. Furthermore, as there is no diagnostic code in the ICD-9 or ICD-10 for MIRM, these patients would likely have been, and continue to be, assigned codes for SJS, TEN, or erythema multiforme with unreliable assignment with secondary codes for mycoplasma infection.

### Proposed revised classification

Author MLR is part of a group that recently proposed a revised classification for severe blistering cutaneous reactions in children; the revision condenses SJS and TEN into a single category of drug-induced epidermal necrolysis (DEN) that may have variable skin involvement (manuscript under revision). This is logical because SJS and TEN are considered quantity variants of the same disease and drugs are their common trigger. The proposed classification separates out the infection-related cases typified by severe mucosal and less impressive skin lesions as reactive infectious mucocutaneous eruption (RIME). Given their disparate causes and pathogeneses, DEN and RIME have diverging management strategies: the focus is on drug withdrawal and early consideration of immunosuppressive treatment in patients with DEN and on identification and treatment of infection combined with supportive care with or without immunosuppressive therapy in RIME cases. Application of this novel pediatric-specific paradigm may have far-reaching impacts on incidence and epidemiology and ultimately provide more directed and effective management.

Biomarkers will likely emerge as tools to confirm diagnosis; the granulysin rapid test, which has a turnaround time of 15 minutes, was used in a 2011 report to establish an early diagnosis of SJS in a young child ^18^. Other candidate biomarkers, including eosinophilia, perforin, interferon-gamma, soluble Fas ligand, and CD69, are being evaluated, although none is validated for clinical use at this time ^19^.

## Etiologies

Recent pediatric retrospective studies identify a drug as the cause of SJS and TEN in 72 to 90% of cases ^20– 23^. Idiopathic cases, where no cause (medication or infection) can be identified, make up 5 to 17% of cases in retrospective reviews ^20, 21, 24^. A limited number of medications, including antibiotics, anticonvulsants, and non-steroidal anti-inflammatory drugs, are implicated in the majority of pediatric cases. As stated above, MP is a common trigger of severe mucocutaneous reactions that have been considered SJS-TEN in the past; however, the clinical presentation and outcomes suggest a unique disease process.

## Causality and risk reduction

It is important to avoid implicating medications used to treat the prodromal symptoms of SJS and TEN as causative agents ^19^. Survivors of SJS and TEN are often told to avoid all medications taken just prior to the reaction, limiting treatments that can be used for future illnesses ^25^ and causing patients and families concern about the use of medications in general. Evaluation of possible implicated medications requires utilization of an effective causality tool. Many available ADR causality tools, including the Naranjo and Liverpool tools, are non-specific to the ADR phenotype. The ALDEN is specific for severe cADRs. These tools are helpful when considering the timing of drug exposure to reaction onset, the probability of drug presence in the body, prior exposure to the same drug regardless of reaction at that time, the presence of drug beyond the progression phase, drug notoriety as an ADR cause, and the presence or absence of other etiologies ^25^. Although these tools can be helpful, their usefulness is limited by variability in results between users and testing methods. Further advancements in diagnostics and assessment tools have the potential to enhance causality assessment in the future ^26^.

Testing to identify the causative agent in SJS-TEN remains controversial and is not widely used. *In vivo* (varied methods of re-exposure of the patient to potential trigger medications) testing includes patch testing or delayed intradermal testing. These tests can be performed once the acute reaction has resolved but within a year. Potentially cross-reacting medications can also be identified with patch testing ^27^. Unfortunately, the results are not reliable for all medications, limiting their clinical utility. Oral re-challenge, recommended for other types of drug reactions, is not recommended for SJS and TEN given the serious risk of a second potentially fatal episode ^28^. *In vitro* testing with the lymphocyte transformation test (LTT) during the recovery phase, 4 to 8 weeks after the reaction, is controversial as LTT historically has had low sensitivity in SJS-TEN and many false-positive and -negative results ^29^. Recent reports support better sensitivity (86%) and specificity (74%) in SJS-TEN, even for low-risk drugs ^30^. The drug-specific interferon-gamma–releasing cell assay is highly specific (95% specificity and 79% sensitivity for allopurinol) and can be carried out in the acute phase ^31^. A recent publication highlighted its advantages over LTT: higher rates of causative drug identification (73.9% versus 52.2% for LTT) and use during the acute phase (versus recovery phase for LTT) ^32^.

Prevention of SJS and TEN is becoming a reality with the identification of risk factors that can be screened prior to drug initiation. Human leukocyte antigen (HLA) screening for ethnicity-specific risk alleles prior to administration of aromatic anticonvulsants (including carbamazepine), allopurinol, and abacavir reduces the risk of SJS-TEN ( Table 4) ^33^. Polymorphisms that reduce drug clearance can also increase the risk of severe cADRs ^34^ When a risk allele is identified in a patient, the medication should be avoided ^35^. As a proof of concept, in Taiwan, where pre-carbamazepine screening for HLA-B*1502 was adopted in 2010, the incidence of SJS-TEN has drastically decreased ^36^. In Thailand, where HLA-B*1502 pre-treatment screening is also routine, HLA test results are printed on a wallet card that patients can carry with them as a “pharmacogenomic ID card” for future health-care interactions ^35^.

**Table 4.** Medication causes of SJS-TEN with strongly predictive (100%) HLA associations ^35^.

Medication	Population	HLA type	Interpretation of positive result
Allopurinol	Han Chinese and European	B*5801	Increased risk of SJS-TEN, do not use in naïve patients, can be considered in patients without reaction after more than 3 months
Carbamazepine	Han Chinese, Thai, Malaysian, Indian, Singaporean, and Vietnamese	B*1502	Increased risk of SJS-TEN, do not use CBZ or ox-CBZ in naïve patients, can consider using if no reaction after more than 3 months treatment
	European, Japanese, and Korean	B*3101	

CBZ, carbamazepine; HLA, human leukocyte antigen; ox-CBZ, oxcarbazepine; SJS-TEN, Stevens–Johnson syndrome – toxic epidermal necrolysis. ^35^. Adapted with permission from Peter *et al*. ^19^.

## To treat or not to treat, that is the question… but we still don’t know the answer

SCORe of Toxic Epidermal Necrolysis (SCORTEN), a TEN severity-of-illness score ^37^, was developed for adults but has been used to predict both morbidity and mortality in pediatric patients ^38, 39^. The SCORTEN has multiple factors, but three of them—age of at least 40 years, malignancy, and heart rate of at least 120 beats per minute—are less important in children because (1) they are young, (2) they have low rates of malignancy, and (3) their normal heart rate can be over 120 beats per minute if they are younger than four. The SCORTEN, or a pediatric-adapted scoring system, could be used prospectively in studies to predict treatment efficacy by comparing actual with predicted mortality and morbidity ^38^. Despite the availability of a validated severity score that helps to predict which patients will have more severe outcomes, the absence of a gold-standard effective therapy for pediatric SJS-TEN means that the decision between supportive care and directed (immunosuppressive) therapy remains challenging.

In the last two years, there has been an explosion of retrospective and database publications on pediatric SJS-TEN which have come to similar conclusions on the relative lack of efficacy of corticosteroids and intravenous immunoglobulin (IVIG) on various outcomes ^20, 22, 40– 43^. For treatment of SJS-TEN, there is a consensus on the importance of rapid identification and withdrawal of the causative medication. As adjuvant therapy, corticosteroids are most frequently used, followed by IVIG both as monotherapy and in combination with corticosteroids. None of these mono- or combination therapies appears to affect time to healing or length of hospital stay. The best evidence on treatment before 2017–2018 was from a large (n = 128 cases) systematic review published in 2011 that suggested that patients who received IVIG and prednisone had better outcomes than those who received supportive care only ^44^. Overall mortality rates for SJS-TEN in newer publications range from 0 to 6.6%, and rates of up to 25% were reported for TEN cases ^20, 22, 40– 43^.

Recent case reports and case series document the rapid efficacy of cyclosporine 3 mg/kg per day divided twice a day and infliximab 5 mg/kg intravenously for one dose in both drug- and infection-related cases ^45, 46^. There is emerging evidence from case reports for anti-tumor necrosis factor (anti-TNF) therapy in the acute phase ^47– 50^. Furthermore, a recent Taiwanese randomized clinical trial of etanercept in TEN included children older than 4 years of age and showed that etanercept (25 mg twice weekly <65 kg and 50 mg if >65 kg) decreased the predicted mortality rate and reduced skin healing time compared with corticosteroids (1 to 1.5 mg/kg per day intravenously) in the group as a whole ^51^. Both groups received treatment until their skin lesions healed ^51^. This study included five children who were 6 to 13 years old ^51^. Further large-scale studies are needed to confirm these promising results in the pediatric population.

Ocular complications and sequelae are significant for pediatric patients with SJS-TEN. Aggressive initial management, including adjuvant amniotic membrane transplantation, can reduce complications ^52, 53^. Even in the absence of severe ocular involvement in acute SJS-TEN, children may develop progressive disease of the ocular surface and conjunctival inflammation over time ^54^. Delayed lid margin keratinization ^55^, vision deterioration, and corneal damage can occur after SJS-TEN ^56^, emphasizing the importance of close continual follow-up by ophthalmology ^55, 57^.

The early introduction of psychological and social support is critical for pediatric patients with SJS-TEN to avoid long-term anxiety and depression. Ideally, the introduction should occur as soon as the patient is stable enough for a psychologist or child-life worker to visit. For children, the experience of complete loss of control of their body can be devastating, and early explanation and attention to the patient’s emotional needs are critical. Other chronic sequelae are summarized in Table 5.

**Table 5.** Potential chronic sequelae of Stevens–Johnson syndrome – toxic epidermal necrolysis.

Organ system	Sequelae
Skin	Dyspigmentation, eruptive nevi, milia, nail dystrophy, alopecia, scarring, and heterotopic ossification
Ocular	Sicca symptoms, trichiasis, corneal vascularization, corneal scarring, symblepharon, keratitis, and blindness
Oral	Xerostomia, synechiae, chronic gingivitis, dental caries, periodontal disease, taste abnormalities, abnormal dental development, and candidiasis
Gastrointestinal	Pancreatitis, colon necrosis, esophageal stenosis and webs, microstomia, and persistent intestinal ulcers
Genitourinary	Vaginal stenosis, labial fusion, hydrocolpos, hematocolpos, dyspareunia, vaginal dryness, and urethral stenosis
Pulmonary	Chronic obstructive bronchitis and bronchiolitis, bronchiectasis, and pharyngeal and laryngeal scarring
Autoimmune	Sjögren syndrome, systemic lupus erythematosus, and autoimmune thyroiditis
Psychiatric	Anxiety and depression

Adapted with permission from Peter *et al.*, ^19^.

### Treatment of reactive infectious disease: *Mycoplasma pneumoniae* and other infections

Given the limited data on reactive infectious mucocutaneous eruptions, including MIRM and non-MP, it remains unclear whether treatment should be similar to that of SJS-TEN. A systematic review by Canavan *et al*. suggests that the majority of patients receive antibiotics and a smaller percentage receive corticosteroids, IVIGs, or supportive care only ^14^. Although antibiotics are used to treat MP respiratory infections, the effect of antibiotics on the skin and mucous membrane changes of MIRM is unclear ^14^. Recurrent disease is reported in up to 20% of patients ^23^, suggesting a genetic susceptibility to reactive mucositis and rash which has yet to be elucidated.

MP-related SJS-TEN overlap has been reported in one pediatric patient to have rapidly re-epithelialized with cyclosporine 3 mg/kg per day over a period of 7 days without co-treatment for MP infection ^45^. A second case report of TEN induced by MP responded rapidly with complete resolution within a week after a single dose of infliximab 5 mg/kg per day (the patient also received meropenem) ^46^. A case series of three children whose MIRM was treated with cyclosporine suggests that the addition of cyclosporine 3 to 5 mg/kg per day for early cases may accelerate resolution compared with supportive care and antibiotics alone ^58^.

Generally, the course of MIRM is less severe than for drug-related SJS-TEN, and supportive care is a reasonable option. However, the mucous membranes should be closely monitored as the same sequelae as are seen in SJS-TEN occur and can be severe. A recent publication in the ophthalmology literature suggests that these children should be followed closely during their inpatient admission similarly to SJS-TEN ^59^. In their systematic review, Canavan *et al*. reported ocular sequelae in 9%, post-inflammatory dyspigmentation in 6%, and oral or genital synechiae in less than 1% ^14^. Severe gynecologic sequelae requiring surgery have been reported ^60^. Given the sequelae and the acute severe disease course, psychosocial support should ideally be introduced to patients and families at the time of diagnosis (as for SJS-TEN) and there should be a regular review for signs of (latent) distress at follow-up visits to their primary care provider.

The bottom line for therapy is that the identified triggering medication should be discontinued, an infection should be sought and treated if suspected, supportive care in a low-ratio nursing environment should be provided, and anti-inflammatory immunosuppressive therapy, particularly in the early acute phase, should be considered. Specialist input should be sought and coordinated. An overview of the approach to practical management of pediatric SJS-TEN is presented in Table 6 and is detailed by McPherson *et al*. ^62^.

**Table 6.** Practical management of pediatric Stevens–Johnson syndrome – toxic epidermal necrolysis.

Admission	Determine cause based on drug history (ALDEN), infectious symptoms **Baseline:** Investigations: confirm cause, rule out contraindications to treatment - routine bloodwork, including complete metabolic profile, liver function tests, urinalysis - infectious workup, including viral serologies/PCR (Epstein–Barr virus, cytomegalovirus, HSV, human herpes virus 6), nasopharyngeal swab for respiratory viruses and *Mycoplasma pneumoniae* PCR, oral mucosal swab for HSV PCR, chest x-ray to rule out pneumonia - screen for HLA risk alleles if not already known ( Table 4) - if patient severe and might need immunosuppression: consider interferon-gamma release assay for tuberculosis, hepatitis and HIV serology, *Strongyloides serology* Document severity: SCORTEN, BSA, photography **Treatment:** Discontinue potential causative medications Treat for infection if present with directed antibiotics Supportive care: sterile wound care, fluid replacement and nutritional supplementation as for burns, airway management, pain control **Plan:** Assess need for transfer to specialized experienced center for severe cases (SCORTEN >1, BSA >10%, comorbidities, requiring ventilation) Consult dermatology, ophthalmology, gynecology, urology, infectious disease, pharmacy/clinical pharmacology urgently Consider anti-inflammatory/immunosuppressive treatment: consider contraindications, risk-benefit
Monitoring	Frequent vital signs, monitor for fever Frequent swabs to identify infection early, prophylactic antibiotics not recommended Document progression with SCORTEN, photography Supportive care, including early physiotherapy
Follow-up	Identify a primary contact for the patient after discharge, either a pediatrician or specialist amongst the following: • Dermatology • Ophthalmology • Gastroenterology • Gynecology (female) and urology (male) • Psychiatry/Psychology for post-traumatic stress disorder • Genetics to review HLA testing and counsel family • Respirology if needed Consider *in vitro* testing with lymphocyte transformation test or ELISpot (controversial) Give patient a wallet card that identifies their history of SJS-TEN and HLA screening result for future medical encounters. Please refer to Figure 4 in Sukasem *et al*. ^35^ for an example.

ALDEN, algorithm of drug causality for epidermal necrolysis; BSA, body surface area; HLA, human leukocyte antigen; HSV, herpes simplex virus; PCR, polymerase chain reaction; SCORTEN, SCORe of Toxic Epidermal Necrolysis; SJS-TEN, Stevens–Johnson syndrome – toxic epidermal necrolysis. ^61, 62^.

## How redefining diagnosis of pediatric Stevens–Johnson syndrome – toxic epidermal necrolysis can improve treatment

The literature on the treatment of pediatric SJS and TEN is disappointing as larger studies suggest no impact of any intervention other than drug withdrawal. In contrast, case series and reports document the effectiveness of various anti-inflammatory immunosuppressive treatments. Could it be that the way that past cases are classified results in groups that are too heterogeneous (that is, triggered by infection or drugs or idiopathic) to respond similarly to a given therapy? Considering a shift in diagnostic paradigm as discussed above may be a way to interpret the existing literature with a lens focused from cause/trigger to treatment. Adding to this a precision medicine approach that compares subpopulations of responders with non-responders to identify characteristics that permit early recognition and takes advantage of biomarkers like the granulysin rapid test may lead to a future in which we can select the right treatment for the right patient every time.

## Conclusions

The future is bright for pediatric SJS-TEN. Initiatives are under way to improve our understanding of this spectrum of disorders, specifically in children. A British guideline on management was recently published ^61^. With increasing accessibility and validation of risk factor screening (HLA and metabolism variants) and biomarkers, we may soon be able to prevent SJS-TEN in predisposed individuals and diagnose and treat it early when it occurs by chance.

## Abbreviations

ADR, adverse drug reaction; ALDEN, algorithm of drug causality for epidermal necrolysis; BSA, body surface area; cADR, cutaneous adverse drug reaction; DEN, drug-induced epidermal necrolysis; HLA, human leukocyte antigen; ICD, International Classification of Diseases; IVIG, intravenous immunoglobulin; LTT, lymphocyte transformation test; MIRM, *Mycoplasma pneumoniae*–induced rash and mucositis; MP, *Mycoplasma pneumoniae*; RIME, reactive infectious mucocutaneous eruption; SCORTEN, SCORe of Toxic Epidermal Necrolysis; SJS, Stevens–Johnson syndrome; TEN, toxic epidermal necrolysis
